# Parafoveal processing of orthographic, morphological, and semantic information during reading Arabic: A boundary paradigm investigation

**DOI:** 10.1371/journal.pone.0254745

**Published:** 2021-08-02

**Authors:** Ehab W. Hermena, Eida J. Juma, Maryam AlJassmi

**Affiliations:** Department of Psychology, Cognition and Neuroscience Research Laboratory, College of Natural and Health Sciences, Zayed University, Dubai, UAE; Basque Center on Cognition Brain and Language, SPAIN

## Abstract

Evidence shows that skilled readers extract information about upcoming words in the parafovea. Using the boundary paradigm, we investigated native Arabic readers’ processing of orthographic, morphological, and semantic information available parafoveally. Target words were embedded in frame sentences, and prior to readers fixating them, one of the following previews were made available: (a) Identity preview; (b) Preview that shared the pattern morpheme with the target; (c) Preview that shared the root morpheme with the target; (d) Preview that was a synonym with the target word; (e) Preview with two of the root letters were transposed thus creating a new root, while preserving all letter identities of the target; (f) Preview with two of the root letters were transposed thus creating a pronounceable pseudo root, while also preserving all letter identities of the target; and (g) Previews that was unrelated to the target word and shared no information with it. The results showed that identity, root-preserving, and synonymous preview conditions yielded preview benefit. On the other hand, no benefit was obtained from the pattern-preserving previews, and significant disruption to processing was obtained from the previews that contained transposed root letters, particularly when this letter transposition created a new real root. The results thus reflect Arabic readers’ dependance on morphological and semantic information, and suggest that these levels of representation are accessed as early as orthographic information. Implications for theory- and model-building, and the need to accommodate early morphological and semantic processing activities in more comprehensive models are further discussed.

## Introduction

Arabic is a Semitic language that is read from right to left. It features Semitic morphology where words are built from non-concatenated combination of root and pattern morphemes. That is, the root letters are typically diffuse within the word and can be interrupted by inserting letters from the pattern morpheme (so-called infixes, e.g., مكتوب /mktub/ *is written*, where the root كتب /ktb/ is interrupted by the letter و /u/ of the pattern morpheme /مـ ـ ـ و - / /m_ _ u _/, see e.g., [1–3]). The root morpheme indicates the main semantic family to which the word belongs (e.g., in the example above, the root كتب /ktb/ refers predominantly to writing-related meanings), whereas the pattern morpheme provides the detailed phonological and syntactic information that are necessary for complete and accurate word identification (e.g., [2]). Therefore, the tightly-knitted orthographic, morphological and semantic information within words in Arabic make it a very interesting medium to investigate how this information is extracted from the parafovea during sentence reading.

### A brief overview of ortho-morphological and semantic processing in Arabic

In alphabetic orthographies, like English and other European languages, evidence shows that letter identity and position information are important variables in word identification. Evidence from transposed letter (TL) studies suggested that these two variables are encoded independently during early lexical processing. Typically, primes with transposed letter order (thus preserving letter identity but not letter position information) result in facilitation in identifying the target word comparable with identity primes (e.g., *JUGDE* as a prime for *judge*), suggesting that there is a good deal of flexibility in letter position encoding. In single word paradigms, TL prime benefits are widely reported in languages such as English, French, Dutch, and Spanish [4–14]. In these languages morphology is sequential and concatenated in nature, and lexical organization is believed to be orthography-based, with entries that share letters, or are orthographic neighbors being clustered together in the lexicon (see [15] for review). Findings from these European languages informed the construction of multiple word identification models, where flexible letter position encoding was thought to be a fundamental feature of the cognitive system (e.g., SOLAR model [16]; Spatial Coding model [17–19]; the SERIOL model [20–23]).

Findings from Semitic languages such as Arabic and Hebrew did not, however, conform with these findings. The root morphemes in Semitic words were found to play an important role in Arabic word identification and in lexical organization, with words that share the same root being clustered together [15,24–35]. For example, primes that shared root information with the target resulted in faster identification of the target, regardless of whether the semantic relationship between the prime and target was transparent (e.g., كتابة /kitaab^a^t/ *writing* and كاتب /kat^i^b/ *writer*), or not (e.g., كتيبة /k^a^t^i^b^a^t/ *squadron* and كاتب /kat^i^b/ *writer*, see [1,36,37]). Importantly, robust findings showed clearly that transposing root letter order in primes (thus preserving root letter identities, but not their order), did not yield a processing benefit as was found in European languages [33,38–40]. Rather, significant processing costs were reported, especially when transposing the root letters created another existing real root, the activation of which through priming is thought to interfere with processing the actual root present in the target [41]. In Arabic transposing root letters results in creating new roots that exist in the lexicon around 54% of the time (e.g., the root بدل /bdl/ *switched* becomes بلد /bld/ *dulled*/*numbed* with transposing the second and third letters, see [15,29,38] for reviews). By contrast, transposing letters in English was estimated to result in creating new words on only 7% of the time [38]. The rigidity in letter position encoding in Semitic languages, compared to European languages, is thus understood to be a product of the orthographic density of Semitic languages ([15] for review).

With regards to word patterns, as mentioned earlier, Arabic words feature pattern morphemes that are interwoven, or infixed into the letters of the root morpheme. Compared to word roots, the exact role of word patterns in word identification, and the time course of processing word patterns remains to be fully delineated. In Hebrew, for instance, whereas verbal word patterns resulted in priming facilitation, nominal word patterns did not [32]. This was attributed to the fact that whereas Hebrew has over one hundred nominal word patterns [28], there are only seven verbal patterns [42], with the smaller number of the latter helping narrow down potential word candidates. On the other hand, investigations of pattern processing in Arabic reported benefit from both verbal and nominal pattern-preserving primes [1,2,36,43,44]. Furthermore, the benefit from pattern-preserving primes in Arabic were found to be influenced by variables such as stimulus onset asynchronies (SOA, or the delay between the onset of the masked prime and the onset of the target, [2]). Interestingly, the benefit from pattern-preserving primes in Arabic were also found to be influenced by root-related variables such as the presence of a transparent semantic relation between the root of the prime and the target; and the productivity (family size) of the root of the target word [44]. Boudelaa and Marslen-Wilson [44] suggested that the difference in how root and pattern information are processed may be influenced by the fact that the Semitic consonantal roots in Arabic are always fully specified, that is, present in print. By contrast, pattern information is mostly only partially specified, given that pattern morphemes often incorporate vowels that are typically represented by diacritics, and that these diacritics are typically omitted from print [45].

Arabic readers were also found to extract semantic information from primes such that facilitation for processing target words was obtained when these targets were preceded by semantically-related primes (e.g., سرير *bed* as prime for وسادة *pillow*) relative to when the prime and target were not semantically related (e.g., عسكري *military* as prime for وسادة *pillow*, see [46,47]). Interestingly, in the task presented by Mountaj et al. [46] the prime remained on the screen for 500 ms. After another 300 ms blank screen, the participants were required to decide whether the prime and target were semantically related. Thus, more remains to be learnt about whether Arabic readers extract meaning from upcoming words during sentence reading (i.e., in time intervals that are typically shorter than what was allowed by Mountaj et al.).

### Parafoveal processing in sentence reading

#### I. Preview benefit and processing of orthographic information in the parafovea

A great deal of evidence from eye movement investigations has established that during a fixation, readers process the fixated word as well as pre-process the upcoming word in the parafovea. Parafoveal pre-processing is thought to resemble the partial processing that takes place in priming [48–51]. The boundary paradigm (Rayner, 1975) [53] is typically used to investigate the extent to which readers extract information from the upcoming word. In this paradigm, an invisible boundary is inserted in the text immediately before a target word. Prior to crossing this boundary, the reader is presented with a preview of the upcoming word that may, or may not, be identical to the target word, or that may share certain linguistic characteristics with it (e.g., orthographic, *bench* as preview of *beach*). As the reader’s eyes saccade across that boundary, forward into the text, the display changes and the target word is displayed correctly when the reader fixates it. Importantly, the reader is typically unaware of the display change because of the suppression of visual processing during saccades [52]. Available evidence shows that when readers are given a valid (i.e., identical) parafoveal preview of the upcoming word, fixation durations on this word are reduced—the so-called *preview benefit*, compared to when the previews are not valid (e.g., the string *dmaeb* as a preview of *beach*, e.g., [53,54]. Schotter [50] suggested that preview benefit effects are due to costs to processing being less severe when the preview and target are similar, that is, share relevant sublexical information, compared to when they are dissimilar. Indeed, investigations in many languages reported that giving readers parafoveal previews that share orthographic and/or phonological information with the targets results in preview benefit, relative to when previews lack such information (e.g., [55–60]).

#### II. Morphological processing in the parafovea: The case of Semitic roots and patterns

In studies that aimed to investigate readers’ parafoveal processing of morphology, readers are typically presented with previews that shared a morpheme with the upcoming target word. In English and other European languages, the results showed no greater benefit from morphologically-related previews relative to orthographic control previews that shared as many letters with the target (see [61–63], and [64] for review). By contrast, findings from parafoveal processing of Hebrew morphology reported that previews that shared the Semitic root with the target resulted in processing benefit (i.e., reduced fixation durations) for the target, compared to orthographic control previews [28,65].

Furthermore, additional findings further supported the idea of rigid letter position encoding in Semitic languages. Specifically, in non-Semitic languages, parafoveal previews that featured transposed letters of the target word (e.g., *cpatain* as preview of *captain*) yielded the typical preview benefit once that target word was fixated (see e.g., [6,66]; and also in children reading [67]), akin to what was reported in single word paradigms (see above). By contrast, and in line with the above discussion, disrupting the order of the Semitic root letters in the parafoveal preview did not lead to processing benefit, rather, it results in inflated fixation durations on the target words [68].

Parafoveal processing of Semitic word patterns was also investigated. Deutsch, Frost, Pollatsek, and Rayner [69] reported facilitation in Hebrew word identification when the parafoveal preview shared the pattern morpheme with the target. However, this was only the case for verbal, not nominal, patterns, thus replicating the findings discussed above from single word processing tasks in Hebrew. However, a more recent investigation using both the boundary paradigm and fast priming techniques, Deutsch, Velan, and Michaly [70] reported that readers do benefit from nominal word patterns in Hebrew. The recorded facilitation represented a subtle effect that took place early during word identification (on average 8 ms in first fixation duration and 17 ms in gaze duration).

#### III. Processing of Semantic information in the Parafovea

Semantic processing was typically presumed to take place later, subsequent to processing other word representations (e.g., orthography and phonology). Given this, and because parafoveal processing, by definition, takes place very early prior to fixating the target word, the absence of evidence for semantic preview benefit in studies of parafoveal processing was not considered surprising [71–74]. Later studies, however, found evidence for semantic preview benefit in German and in Chinese, where semantically related previews resulted in more facilitation in target word processing relative to previews that were not semantically related to the target [75–78]. Differences in orthographic transparency between the languages (German being more transparent in its letter-sound correspondences than English), and the nature of ortho-semantic relationship (being closer for Chinese compared to English, with the semantically transparent Chinese radicals) were suggested as potential explanations for German and Chinese readers’ ability to access semantic information parafoveally. By contrast, English readers were suggested not to reach this depth of processing until later stages of word identification [51,79].

In later investigations, researchers reported that readers of English also perform parafoveal semantic processing. In one investigation, preview benefit was obtained from synonymous parafoveal previews (e.g., *video* as preview of *movie*, [80]), while no significant benefit was obtained from previews that were only semantically related to the target (e.g., *audio* as preview of *movie*). The benefit obtained from the closeness of the semantic relationship between the preview and target in English was found to be modulated by visual and orthographic word-level variables such as initial letter capitalization in the previews [81]. This benefit was also found to be modulated by sentence level variables such as the degree of sematic constraining of pre-target context (e.g., the word *scrub* after the neutral context “My roommate will continuously …” vs. the constraining context “For hygienic purposes, doctors must …,” see [82]); and the plausibility of the preview in the sentence context (e.g., plausible “… scared that a freak *twister*/ *tsunami* …” vs. implausible “… scared that a freak *booster* …”, see [83–85], also [78,86] for similar findings in Chinese). As yet, processing semantic information parafoveally during reading Arabic has not been investigated.

### The current research

In the study reported here we aimed to examine the parafoveal processing of orthography, morphology and semantics in Arabic sentence reading. As illustrated above, the tight knitting of these levels of representation in Arabic words makes it a fertile medium for studying readers’ access and processing of the information conveyed in printed text in a comprehensive way. There are numerous outstanding questions regarding the extent, time course, and effects of accessing various ortho-morphological, and semantic information parafoveally. Specifically, we aimed to investigate the following: (a) Parafoveal processing of Arabic orthography: Replicating the basic findings that identity previews result in preview benefit, relative to orthographically unrelated previews; (b) Parafoveal ortho-morphological processing: The effects of transposing root letters in the parafoveal preview, when the letter transposition creates a new (real) root, and when it creates a pseudo root (pronounceable but meaningless), while preserving letter identities in both cases; (c) Morphological parafoveal processing: The effects of providing readers with parafoveal previews that preserve the root morpheme, and previews that preserve the pattern morpheme; and finally, (d) Semantic processing in the parafovea: Exploring whether readers would obtain preview benefit if the preview was a synonymous word with the target. The experimental conditions and statistical contrasts we utilized to investigate these questions are detailed in the following sections.

Based on the previous findings discussed above, we expected to obtain the classic preview benefit for identity previews, and benefits from root-preserving previews. Similarly, we expected that transposing root letters in the previews would result in processing costs, particularly when the letter transpositions create a new real root, given the rigid letter position encoding in Semitic languages (e.g., [15,29,38]). By including both TL preview conditions, however, we aimed to learn more about the costs of creating a TL real root—a lexical competitor to the root embedded in the actual target, compared to a TL pseudo root that arguably does not generate lexical competition to the root embedded in the actual target. Additionally, we had no clear expectations for the effects of pattern-preserving previews, given the inconsistent findings obtained so far in silent reading and single word tasks. Indeed, pattern-based processing facilitation reported in studies of Hebrew [69–70] have yet to be replicated in Arabic, where, so far the reported effects of preserving word patterns seem to be modulated by experimental variables (e.g., SOA, see [2] and discussion above), and root-related variables (e.g., root productivity, see conclusions in [44]). Similarly, we had no clear expectations of whether previews that are semantically identical to the targets (i.e., synonyms) would yield preview benefit. However, in the light of previous findings, it is plausible that Arabic readers may extract semantic information parafoveally, perhaps by virtue of the transparency of the Arabic consonants’ letter-sound correspondences that would facilitate earlier access to meaning (e.g., [80]).

## Method

### Participants

A total of seventy-seven adult native Arabic readers, all undergraduates at Zayed University, UAE, took part in the eye tracking procedure (M = 21.4, SD = 2.1, Range = 18–24). All participants had normal or correct to normal vision as determined by the Bailey-Lovie chart [87]. Participants received AED20 (~ $5.30) coffeeshop vouchers as incentive for participation.

For stimuli norming (more details below), a total of 15 participants were recruited from the same population (undergraduate, native Arabic readers). They also received AED20 coffeeshop vouchers for participation. These participants, however, did not take part in the eye tracking procedure.

### Stimuli

**Fig 1:**
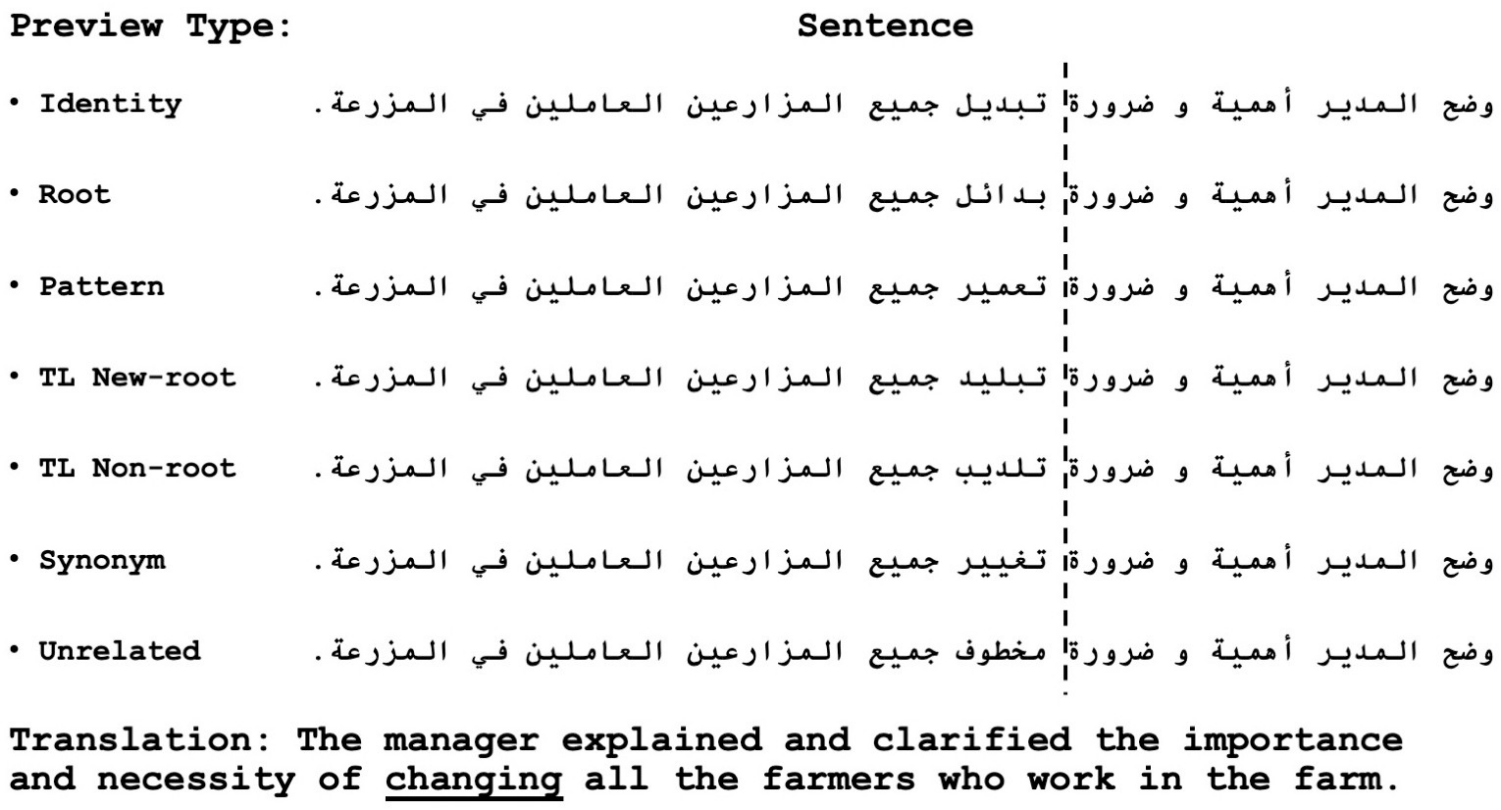
Sample stimuli set. The dashed line indicated the location of the invisible boundary, followed by the different preview strings in the different conditions.

Sixty-three target words were embedded in frame sentences (see Fig 1 for an example). The target words were 4–8 letters long (M = 5.4, SD = 1.1) and each contained a 3-letter root. For the target words, and all words used as previews, Table 1 provides orthographic frequency (counts per million, CPM) in the Aralex database [88], as well as ratings of words’ and roots’ commonness of occurrence in the language. The commonness of occurrence ratings for each word and each root were collected from 5 participants who did not take part in the eye tracking procedure on a 5-point scale (1 indicating very rare occurrence, 5 indicating very common occurrence).

**Table 1:** Preview conditions details and an example.

Preview Condition	Example	Translation	Lexicality	Average Number Shared Letters with Target (SD)	Average Word Frequency CPM in Aralex (SD)	Average Root Token Frequency CPM in Aralex (SD)	Average Pattern Token Frequency CPM in Aralex (SD)	Average Ratings of word Commonness of Occurrence (SD)	Average Ratings of Root Commonness of Occurrence (SD)
Identity	تبديل/tbdil/	*Switching/Change*	Word	All	12.6 (40.4)	741.4 (1070.0)	15307.1 (11559.6)	4.1 (0.6)	4.4 (0.7)
Pattern	تعمير/tʕmir/	*Constructing*	Word	2.6 (1.1)	29.7 (77.1)	1059.1 (1380.7)	15307.1 (11559.6)	3.9 (0.7)	4.3 (0.6)
Root	بدائل/bdaal/	*Alternatives*	Word	3.6 (0.7)	6.2 (13.4)	741.4 (1070.0)	16594.7 (11966.0)	4.2 (0.5)	4.0 (0.6)
TL New Root	تبليد/tblid/	*Dulling/Numbing*	Word	All	8.7 (23.9)	517.7 (837.1)	15307.1 (11559.6)	4.2 (0.7)	4.5 (0.7)
TL Non-root	تلديب/tldib/	*NA*	Pseudo Word	All	NA	NA	15307.1 (11559.6)	NA	NA
Synonym	تغيير/tgyir/	*Change*	Word	2.9 (1.2)	13.2 (38.1)	953.8 (1559.0)	15449.9 (12669.6)	3.9 (0.8)	4.4 (0.5)
Unrelated	مخطوف/mxtuf/	*Kidnapped [noun]*	Word	0	9.4 (22.6)	667.9 (1112.1)	14633.1 (14646.7)	4.0 (0.7)	4.3 (0.7)

The root letters in the Example column are underlined for illustration. SD = standard deviation, CPM = raw counts per million in the Aralex corpus (Boudelaa & Marslen-Wilson, 2010 [88]).

To allow us to answer the research questions detailed above, seven parafoveal preview conditions were created for each of the target words. These were: (a) Identity preview, where the target word itself without any alterations was the preview; (b) Pattern-preserving preview, where the pattern of the target word was preserved and presented in the preview with a new root (29 patters, around 40%, were verbal, and the rest were nominal); (c) Root-preserving preview, where the root of the target word was preserved and combined with a new pattern; (d) Synonym preview, where the preview was a synonym of the target word, without sharing any root information with the target (i.e., the synonym was derived from a different root), the synonyms were chosen from the major nine Arabic language dictionaries (these are:لسان العرب، مقاييس اللغة، الصحاح في اللغة، القاموس المحيط، العباب الزاخر، مختار الصحاح، المعجم الوسيط، تاج العروس، ومعجم اللغة العربية المعاصرة. We used the electronic searchable versions of these dictionaries available at http://www.maajim.com and http://www.baheth.info); (e) TL New Root preview, where the position of two root letters was transposed, creating a new real root in a new real word; (f) TL Pseudo Root preview, where two root letters were transposed, creating a pseudo root, that is, a pronounceable but meaningless root in a pronounceable pseudo word, the created root and word did not correspond to any root entries in any of the major nine Arabic language dictionaries; and finally, (g) an Unrelated preview that shared no information with the target word.

Note that in the two TL conditions, the preview shared all target word letter identities, including the same unaltered word pattern (see e.g., [40]). Preserving the word pattern in the TL conditions meant that the manipulation is not contaminated by crossing morphemic boundaries between root and pattern [89]. Previous investigations reported that transposing non-adjacent letters may result in weakening or delaying the onset of the recorded transposed letter effect [90,91]. However, given the relative shortness of Arabic words compared to other languages (see discussions in [92,93]), we were compelled to use letter transpositions where the transposed letters were adjacent or separated by either one or two letters. Specifically, in the TL New-root condition, 44.4% of the letter transpositions involved adjacent letters, also about 44.4% involved letters separated by 1 letter, and the remaining (11.1%) contained transposed letters that were separated by 2 letters. In the TL Pseudo-root condition, 60.3% of the letter transpositions involved adjacent letters, 23.8% involved letters separated by 1 letter, and the remaining (15.9%) contained transposed letters that were separated by 2 letters.

In all stimuli sentences, the target words (and previews) always occurred near the middle of the sentence and were never the first or last three words of the sentence. The sentences contained, on average 68.7 characters (SD = 11.0). The sentences were displayed on a single line at the center of the monitor.

#### Stimuli matching and norming

The target word and its previews were matched on number of letters and spatial extent [94]. With the exception of the Unrelated and TL Pseudo Root previews, all preview conditions were of the same syntactic case as the target. We aimed to match the Pattern, the Root and Synonym previews, as much as possible, on how many letters they shared with the target (see Table 1). It was inevitable however that Root previews shared more letters with the target than the Pattern or Synonym previews (*F*(2,186) = 14.4, *p* < .001; post-hoc Tukey test for Root vs. Pattern *p* < .001; Root vs. Synonym *p* = .001; and Pattern vs. Synonym *p* = .23). This is because Root previews and the target may by necessity share the root letters plus one more letter that belongs to the pattern morpheme, given the relatively small number of letters that can be part of pattern morphemes [95].

We also aimed to match the target and previews on word, root and word pattern frequencies (based on Aralex database), as much as possible. In addition, we obtained subjective ratings of word and root commonness as additional indicators of frequency for the targets and previews (see Table 1). For all six preview conditions that were real words, there was no significant difference between log-transformed word orthographic frequencies (all *F*s(5,325) < 1.7, all *p*s >.09). For root frequencies, only the difference between the Aralex log-transformed root token frequency of the Identity and TL New Root conditions reached significance (*t* = 3.0, *p* < .01). None of the remaining root token frequency contrasts were significant (all *t*s < 1.4, *p*s >.50). For pattern frequencies, only the difference between the Aralex log-transformed pattern token frequency of the Identity and the Unrelated previews reached significance (*t* = 2.7, *p* < .01). None of the remaining pattern token frequency contrasts were significant (all *t*s < 1.1, *p*s >.29). Importantly, participants’ ratings of commonness of word and root occurrence in the language did not differ significantly between any of the real word preview conditions (all *F*s(5,325) < 1, all *p*s >.80).

As part of the stimuli norming, target and preview words’ predictability was determined using a cloze procedure. Five participants were given the stem sentences up to, and not including, the target word and were asked to produce the word they thought would occur next in the sentence. If the participants produced either the target word or any of its previews, the stem sentence was changed. In the sentences used as stimuli none of the target words or previews were produced by the participants, indicating that none of these words were predictable from the sentence stem (i.e., the target and preview words were produced on zero occasions by the participants). Finally, we obtained ratings of sentence structure naturalness for all target sentences containing the target word on a 5-point scale (1 = structure is highly unusual, 5 = structure is highly natural). 5 ratings per sentence were obtained from 5 participants, and these indicated that sentence structure for all stimuli in all conditions was highly natural with average ratings of 4.1 (SD = 0.5).

### Apparatus

EyeLink 1000+ eye tracker was used to sample readers’ eye movements during reading. Viewing was binocular, but eye movements were recorded from the right eye only. The eye tracker sampling rate was set to 1000Hz. The eye tracker was tower-mounted, and interfaced with a Silverstone computer, and with a 24-inch BenQ monitor. Monitor resolution was set at 1920 × 1080 pixels, with the maximum vertical refresh rate (144Hz) to minimize display refresh time. The participants leaned on a headrest to reduce head movements. The sentences were displayed in black on a light grey background. The participants viewed the screen from 80 cm, and at this distance each character subtended approximately 0.14° of visual angle, and average number of pixels = 9.4 (SD = 1.1).

### Design

The parafoveal preview condition was the within-participant independent variable. The order of sentence presentation was randomized, and the preview condition presentation was counterbalanced such that each participant saw each sentence only once, and only in one of the 7 preview conditions.

### Procedure

This experiment was approved by Zayed University Research Ethics Committee. At the beginning of the testing session, the participants were given the consent form package (including information sheet). Consenting participants subsequently took part in a vision acuity test before taking part in the eye tracking procedure.

The eye tracker was calibrated using a horizontal 3-point calibration at the beginning of the experiment, and the calibration was validated. Calibration accuracy was always ≤ 0.25°, otherwise calibration and validation were repeated. Prior to the onset of the target sentence, a circular fixation target (diameter = 1°) appeared on the screen in the location of the first character of the sentence. When the tracker registered a stable fixation on the circle, the sentence was displayed.

The participants were told to read silently and press a button on the button box when finished reading each sentence. Additionally, they would periodically be required to use the button box to provide a yes/no answer to the questions that followed approximately one-third of the sentences. For the sentence given in Fig 1, for example, the question would be “The manager wanted to reward all the farmers: yes/no.” Before being exposed to the experimental sentences, the participants read 7 practice sentences (3 followed by yes/no questions) to become fully acquainted with the procedure. In total, thus, the participants read 70 sentences (63 experimental sentences + 7 practice sentences).

The participants were allowed to take breaks followed by re-calibration of the tracker. The testing session lasted around 30 minutes, depending on how many breaks a participant took.

## Results

Seven participants were excluded from the analyses given that their sentence comprehension scores fell below 80%. Thus, the reported results are based on data collected from 70 participants. These participants had an average sentence comprehension score of 96% (SD = 3.2, range = 84–100%).

For all reported analyses, fixations with durations shorter than 80 ms, or longer than 800 ms were removed. However, fixations shorter than 80 ms that were located within 10 pixels or less from another longer fixation were merged with the longer fixation. Trials where blinks occurred were removed. In addition, we also removed trials where the display change occurred too early during a fixation on the pre-boundary (pre-target) word, or too late after the onset of a fixation on the target word. These procedures resulted in removing about 4.5% of all data points (a total of 4,211 trials remaining).

From the remaining dataset, to ensure that participants had access to high quality linguistic information in the parafovea, two further steps were implemented. First, only trials where the pre-boundary (pre-target) word was fixated immediately before fixating the target (i.e., not skipped) were included in the analyses. This resulted in removing about 6.2% of all data points (a total of 3,949 trials remaining). Subsequently, we removed all trials where the pre-target word was fixated but the fixation was too far from the target word. Specifically, fixations on the target word that had a launch site of ≥ 65 pixels (around 6.9 characters) from the space before the target word were removed. This resulted in removing around 5.4% of the remaining data points (a total of 3,735 trials remaining). Launch distance is an important variable when investigating parafoveal processing (e.g., [96]). This applies particularly to Arabic script [93,97] given its visual confusability and complexity (e.g., depending on very small, letter-part, identifying features of such as number and location of small dots, or the number of very short vertical strokes, etc.).

We report a number of eye movement measures on the target word region. The first measure is *word skipping probability* (the probability that the target word was not fixated during first pass reading). We also report measures of first pass reading, namely: *First fixation duration* (the duration of the first fixation on the target word, regardless of the number of fixations the word received overall); *single fixation duration* (the duration of the fixation on the target in instances where the target received exactly one fixation during sentence reading); and *gaze duration* (the sum of fixation durations the target word received during first pass reading and before exiting the target word to go forward or backwards in the text). We also report the later measure of *total fixation time* (the sum of all fixation durations the target word received in all passes). In addition, we also report analyses of *spillover*, or the duration of the first fixation on the post-target word, subsequent to fixating the target word. This allowed for determining whether there were any spillover effects of processing the parafoveal previews of the target. Table 2 contains the descriptive statistics for all reported measures for both the target word and the post-target (spillover) regions.

**Table 2:** Descriptive statistics of eye movement measures for the target and post-target regions.

		Preview Condition						
		Identity	Pattern	Root	Synonym	TL New Root	TL Pseudo Root	Unrelated
Interest Region	Eye Movement Measure	Mean (SD)	Mean (SD)	Mean (SD)	Mean (SD)	Mean (SD)	Mean (SD)	Mean (SD)
Target Word	Skipping Probability	0.011 (0.106)	0.004 (0.061)	0.010 (0.097)	0.008 (0.086)	0.004 (0.061)	0.007 (0.086)	0.009 (0.096)
	First Fixation Duration (ms)	278 (118)	318 (142)	279 (118)	283 (105)	360 (149)	328 (149)	320 (147)
	Single Fixation Duration (ms)	281 (108)	337 (143)	285 (117)	294 (107)	404 (142)	345 (141)	337 (142)
	Gaze Duration (ms)	386 (254)	419 (244)	383 (211)	389 (207)	475 (251)	416 (233)	423 (223)
	Total Fixation Time (ms)	505 (365)	570 (378)	503 (314)	514 (363)	608 (343)	559 (397)	573 (395)
Post-Target Word	First Fixation Duration (Spillover, ms)	278 (119)	284 (122)	281 (114)	284 (110)	283 (129)	283 (124)	283 (123)

In order to answer the research questions outlined above, we constructed three contrast matrices. The first two matrices were used as a first-stage analysis to answer the primary research questions. Specifically, in *the first contrast matrix* the Identity preview condition was prespecified as the baseline condition against which all other preview conditions were contrasted. In *the second contrast matrix*, the Unrelated preview condition was prespecified as the baseline condition to be contrasted with the other preview conditions (excluding the Identity preview given that the two conditions have already been contrasted in the first matrix). To explicitly map the contrasts from the first and second matrices on to the specific research questions outlined above: (a) Contrasting the Identity vs. Unrelated previews allowed us to answer the question regarding parafoveal orthographic processing in reading Arabic; (b) Contrasting the two TL conditions vs. both the Identity and Unrelated preview conditions allowed for investigating parafoveal ortho-morphological processing, and if these root letter transpositions in the preview would result in costs to processing the target, thus replicating previous findings; (c) Contrasting the Root and Pattern preview conditions to both the Identity and Unrelated previews allowed for investigating parafoveal morphological processing, namely the effect of the availability of these two morphological units in the parafovea; and finally (d) Contrasting the Synonym preview to both the Identity and Unrelated previews allowed for investigating semantic processing in the parafovea, by exploring whether readers would obtain preview benefit if the preview word was synonymous with the target.

*The third contrast matrix* was built to answer secondary questions through contrasting specific pairs of preview conditions. This matrix contained the following pre-specified contrasts: (i) Root vs. Pattern previews, to compare the effects of preserving these two morphological constituents in the parafoveal preview on target word processing; (ii) Root vs. Synonym previews, to compare the effects of preserving morphological (root, which also preserves *partial* semantic information) relative to preserving the *full* semantic information (synonym) parafoveally; and finally (iii) TL New Root vs. TL Pseudo Root, to investigate the potential costs of the lexical competition resulting from the letter transposition that creates a new existing root, relative to the letter transposition that merely disrupts root letter order, but does not create a lexical competitor.

We used the *lme4* package (version 1.1–23, [98]) within the R environment for statistical computing [99] to analyze the raw fixation duration measures by fitting generalized linear mixed-effects models (GLMMs), with Gamma-distribution assumed for the fixation durations that were the dependent variables. Using GLMMs to analyze raw positively-skewed response times, including fixation durations, maintains the transparency of the reported analyses while satisfying the necessary normality assumptions, without the need to transform data [100]. In these models the preview condition was the fixed factor and subjects and items were the random variables. For word skipping probability we used GLMMs with a binomial-distribution assumed for the dependent variable. We always started by running models with maximal random structure [101]. We trimmed the models when failure to converge occurred, or when singular boundaries (suggesting overparameterization) were identified. All findings reported here are thus from successfully converging models. For each eye movement measure we report beta values (b), standard error (SE), *t* statistic for fixation duration measures, and *z* statistic for skipping probability, and the *p* value associated with the *t* or *z* statistic.

Bonferroni correction was applied to reduce family-wise error rate resulting from running multiple contrasts on the eye movement measures for the analyses conducted at both the target word, and the post-target word regions [102]. For the target word region, the Bonferroni-corrected α = .05 ÷ (5 eye movement measures × 3 contrast matrices) ≤.003. For the post-target word (spillover), the Bonferroni-corrected α ≤.05 ÷ (1 eye movement measures × 3 contrast matrices) = .016.

### I. Parafoveal orthographic processing

#### Target word region

*Identity preview vs*. *Unrelated preview (matrix 1)*. The small difference in skipping probability between these two conditions was not statistically significant. For the fixation durations measures, Identity previews yielded significantly shorter fixation in all reported measures relative to Unrelated previews (Tables 2 & 3). The results thus replicate the classic preview benefit findings for orthographically identical previews, and also indicate that our experimental manipulation is functioning as expected.

**Table 3:** Linear mixed models outputs for the contrast matrix of measures with Identity preview as baseline.

Eye Movement Measure	Contrast Preview vs. Identity Preview	*b*	*SE*	*t/z*	*p*
Skipping	(Intercept)	-4.911	0.565	-8.687	**< .001**
	Pattern	-1.103	0.821	-1.344	.179
	Root	-0.146	0.613	-0.238	.812
	Synonym	-0.389	0.652	-0.597	.550
	TL New Root	-1.130	0.821	-1.377	.169
	TL Pseudo Root	-0.383	0.652	-0.587	.557
	Unrelated	-0.182	0.613	-0.297	.767
First Fixation Duration	(Intercept)	280.382	6.285	44.614	**< .001**
	Pattern	38.914	4.531	8.589	**< .001**
	Root	-0.263	4.858	-0.054	.957
	Synonym	5.619	4.488	1.252	.211
	TL New Root	84.914	5.451	15.579	**< .001**
	TL Pseudo Root	45.800	4.704	9.737	**< .001**
	Unrelated	36.599	5.020	7.291	**< .001**
Single Fixation Duration	(Intercept)	284.590	7.215	39.445	**< .001**
	Pattern	55.781	6.205	8.990	**< .001**
	Root	3.161	5.810	0.544	.587
	Synonym	12.326	5.831	2.114	.035
	TL New Root	125.817	7.030	17.898	**< .001**
	TL Pseudo Root	60.609	5.997	10.106	**< .001**
	Unrelated	50.049	6.314	7.926	**< .001**
Gaze Duration	(Intercept)	386.081	7.032	54.903	**< .001**
	Pattern	37.975	8.779	4.326	**< .001**
	Root	11.059	7.788	1.420	.156
	Synonym	11.005	11.413	0.964	.335
	TL New Root	97.918	13.151	7.446	**< .001**
	TL Pseudo Root	33.881	10.416	3.253	**.001**
	Unrelated	43.001	6.063	7.093	**< .001**
Total Fixation Time	(Intercept)	498.449	27.411	18.184	**< .001**
	Pattern	62.353	18.919	3.296	**.001**
	Root	4.714	18.982	0.248	.804
	Synonym	9.179	18.911	0.485	.627
	TL New Root	101.542	18.796	5.402	**< .001**
	TL Pseudo Root	56.149	18.880	2.974	**.003**
	Unrelated	73.924	18.857	3.920	**< .001**
Spillover	(Intercept)	267.020	6.352	42.034	**< .001**
	Pattern	7.991	5.481	1.458	.145
	Root	8.135	5.426	1.499	.134
	Synonym	6.380	5.584	1.143	.253
	TL New Root	4.907	5.003	0.981	.327
	TL Pseudo Root	9.115	5.345	1.705	.088
	Unrelated	8.723	5.294	1.648	.099

TL = transposed letters of the root of the target word. *p* values are marked in boldface as significant only when Bonferroni-corrected α ≤.003 for the target word, and α ≤.016 for the Spillover measure. Spillover corresponds to the first fixation duration on the post-target word. Final reported models in S1 File.

#### Post-target region

*Identity preview vs*. *Unrelated preview (matrix 1)*. The spillover measure (first fixation on post-target word) did not show a significant difference between these two previews conditions (see Tables 2 & 3). This indicates that the processing cost of the Unrelated previews was incurred only locally on the target word itself.

### II. Parafoveal ortho-morphological processing

#### Target word region

*Identity preview vs*. *TL New Root preview (matrix 1)*. The difference between the two conditions in skipping probability was not statistically significant (Tables 2 & 3). On the other hand, the TL New Root preview condition yielded significantly longer fixation durations in all reported measures relative to the Identity preview. Recall that this TL condition shared all the orthographic (letter identity) information with the target, and differed only in the letter order of root morpheme.

*Unrelated preview vs*. *TL New Root preview (matrix 2)*. The difference in skipping probability between the two conditions was not statistically significant (Tables 2 & 4). On the other hand, the TL New Root preview yielded significantly longer fixation durations in all reported measures relative to the Unrelated preview, indicating a substantial processing cost for the former.

**Table 4:** Linear mixed models outputs for the contrast matrix of measures with unrelated preview as baseline.

Eye Movement Measure	Contrast Preview vs. Unrelated Preview	*b*	*SE*	*t/z*	*p*
Skipping	(Intercept)	-5.366	0.430	-12.472	**< .001**
	Pattern	-0.491	0.576	-0.852	.394
	Root	0.373	0.417	0.895	.371
	Synonym	0.146	0.450	0.323	.746
	TL New Root	-0.518	0.576	-0.899	.368
	TL Pseudo Root	0.153	0.451	0.339	.735
First Fixation Duration	(Intercept)	310.613	8.213	37.819	**< .001**
	Pattern	3.503	3.981	0.880	.379
	Root	-34.420	4.069	-8.458	**< .001**
	Synonym	-28.656	4.196	-6.829	**< .001**
	TL New Root	48.135	4.352	11.061	**< .001**
	TL Pseudo Root	10.114	5.016	2.016	.044
Single Fixation Duration	(Intercept)	328.622	5.721	57.445	**< .001**
	Pattern	4.399	5.313	0.828	.408
	Root	-45.947	4.611	-9.965	**< .001**
	Synonym	-37.005	4.451	-8.314	**< .001**
	TL New Root	70.992	5.620	12.632	**< .001**
	TL Pseudo Root	8.950	5.121	1.748	.081
Gaze Duration	(Intercept)	417.434	5.415	77.090	**< .001**
	Pattern	-2.028	4.996	-0.406	.685
	Root	-30.792	4.287	-7.183	**< .001**
	Synonym	-26.521	4.567	-5.807	**< .001**
	TL New Root	62.074	5.432	11.428	**< .001**
	TL Pseudo Root	-2.460	4.478	-0.549	.583
Total Fixation Time	(Intercept)	561.041	7.281	77.051	**< .001**
	Pattern	5.078	5.966	0.851	.395
	Root	-40.140	5.642	-7.114	**< .001**
	Synonym	-37.250	4.716	-7.900	**< .001**
	TL New Root	71.781	5.710	12.572	**< .001**
	TL Pseudo Root	-0.538	5.956	-0.090	.928
Spillover	(Intercept)	273.485	7.167	38.160	**< .001**
	Pattern	0.457	4.231	0.108	.914
	Root	0.557	4.261	0.131	.896
	Synonym	-1.169	4.235	-0.276	.783
	TL New Root	-2.581	4.070	-0.634	.526
	TL Pseudo Root	1.552	4.220	0.368	.713

TL = transposed letters of the root of the target word. *p* values are marked in boldface as significant only when Bonferroni-corrected α ≤.003 for the target word, and α ≤.016 for the Spillover measure. Spillover corresponds to the first fixation duration on the post-target word. Final reported models in S1 File.

*Identity preview vs*. *TL Pseudo Root preview (matrix 1)*. The difference in skipping probability between the two conditions was not statistically significant (Tables 2 & 3). The TL Pseudo Root preview yielded significantly longer fixation durations in all reported measures relative to the Identity preview. Recall that this TL condition also shared all the orthographic (letter identity) information with the target, and differed only in the letter order of root morpheme.

*Unrelated preview vs*. *TL Pseudo Root preview (matrix 2)*. The differences between the two conditions in skipping probability and all the reported fixation duration measures were not statistically significant (Tables 2 & 4).

*TL Pseudo Root vs*. *TL New Root (matrix 3)*. The difference in skipping probability between the two conditions was not statistically significant (Tables 2 & 5). TL New Root previews, on the other hand, yielded significant increases in all reported fixation durations relative to TL Pseudo Root previews. Overall, thus, these findings suggest a substantial disruption to processing when root letters were transposed and created a new root—a lexical competitor to the root embedded in the actual target, relative to when the root letter transposition does not create such a competitor.

**Table 5:** Linear mixed models outputs for the additional pre-specified contrasts.

Eye Movement Measure	Contrast Preview Conditions	*b*	*SE*	*t/z*	*p*
Skipping	(Intercept)	-5.355	0.429	-12.491	**< .001**
	Root vs. Pattern	-0.497	0.471	-1.055	.291
	Root vs. Synonym	0.135	0.412	0.328	.743
	TL Pseudo Root vs. TL New Root	-0.282	0.359	-0.785	.432
First Fixation Duration	(Intercept)	311.475	4.854	64.166	**< .001**
	Root vs. Pattern	27.425	5.082	5.397	**< .001**
	Root vs. Synonym	-10.601	4.115	-2.576	.010
	TL Pseudo Root vs. TL New Root	15.224	4.179	3.642	**< .001**
Single Fixation Duration	(Intercept)	327.851	5.937	55.225	**< .001**
	Root vs. Pattern	36.168	4.714	7.672	**< .001**
	Root vs. Synonym	-13.107	4.429	-2.959	**.003**
	TL Pseudo Root vs. TL New Root	24.564	3.526	6.966	**< .001**
Gaze Duration	(Intercept)	417.230	6.526	63.933	**< .001**
	Root vs. Pattern	19.264	4.525	4.257	**< .001**
	Root vs. Synonym	-7.219	4.529	-1.594	.111
	TL Pseudo Root vs. TL New Root	28.374	3.934	7.212	**< .001**
Total Fixation Time	(Intercept)	560.591	6.696	83.718	**< .001**
	Root vs. Pattern	32.486	4.895	6.637	**< .001**
	Root vs. Synonym	-14.449	5.944	-2.431	.015
	TL Pseudo Root vs. TL New Root	31.813	5.384	5.909	**< .001**
Spillover	(Intercept)	273.774	6.842	40.016	**< .001**
	Root vs. Pattern	0.443	4.491	0.099	.922
	Root vs. Synonym	-1.939	4.748	-0.408	.683
	TL Pseudo Root vs. TL New Root	-2.380	4.195	-0.567	.570

TL = transposed letters of the root of the target word. *p* values are marked in boldface as significant only when Bonferroni-corrected α ≤.003 for the target word, and α ≤.016 for the Spillover measure. Spillover corresponds to the first fixation duration on the post-target word. Final reported models in S1 File.

#### Post-target region

None of the contrasts listed above yielded statistically significant difference in the spillover measure (see Tables 2–5). This indicates that the processing costs associated with TL previews were incurred only locally on the target word itself.

### III. Parafoveal morphological processing

#### Target word region

*Identity preview vs*. *Pattern preview (matrix 1)*. The difference between the two conditions in skipping probability was not statistically significant. Pattern preview however resulted in significant increase in fixation durations, in all reported measures, relative to Identity preview (Tables 2 & 3).

*Unrelated preview vs*. *Pattern preview (matrix 2)*. There was no statistically significant difference between the two preview conditions in the skipping probability or in any of the fixation duration measures (Tables 2 & 4).

*Identity preview vs*. *Root preview (matrix 1)*. There was no statistically significant difference between the two preview conditions in the skipping probability or in any of the fixation duration measures (Tables 2 & 3).

*Unrelated preview vs*. *Root preview (matrix 2)*. The difference between the two conditions in skipping probability was not statistically significant. Fixation durations on the target following Root previews, however, were significantly shorter, in all reported measures, relative to Unrelated previews (Tables 2 & 4).

*Root preview vs*. *Pattern preview (matrix 3)*. The difference between the two conditions in skipping probability was not statistically significant. Fixation durations on the target following Root previews, however, were significantly shorter, in all reported measures, relative to Pattern previews (Tables 2 & 5). Thus, overall, the results point at preview benefit from Root, but not from Pattern previews.

#### Post-target region

None of the contrasts listed above yielded statistically significant difference in the spillover measure (see Tables 2–5).

### IV. Parafoveal semantic processing

#### Target word region

*Identity preview vs*. *Synonym preview (matrix 1)*. There was no statistically significant difference between the two preview conditions in the skipping probability or in any of the fixation duration measures (Tables 2 & 3).

*Unrelated preview vs*. *Synonym preview (matrix 2)*. The difference between the two conditions in skipping probability was not statistically significant. Fixation durations on the target following Synonym previews, however, were significantly shorter, in all reported measures, relative to Unrelated previews (Tables 2 & 4). This indicates the presence of a preview benefit from Synonym previews.

*Root preview vs*. *Synonym preview (matrix 3)*. The difference between the two conditions in skipping probability was not statistically significant. Fixations durations were on the whole numerically shorter following Root previews compared to Synonym previews. The difference reached statistical significance (at the Bonferroni-corrected α level) only at single fixation duration (Tables 2 & 5).

#### Post-target region

None of the contrasts listed above yielded statistically significant difference in the spillover measure (Tables 2–5).

## Discussion

In the current study we aimed to investigate orthographic, morphological, and semantic parafoveal processing during Arabic sentence reading. The results obtained replicated some classic preview benefit effects, and expanded existing knowledge about parafoveal processing during reading Arabic.

### Parafoveal orthographic processing

The reported results replicated the preview benefit findings (see above) for orthographically identical previews (e.g., [55–59]). Identity previews indeed yielded significantly shorter fixation durations relative to Unrelated previews. In addition to indicating that the experimental manipulation was working as expected, these results clearly indicate that Arabic readers performed parafoveal orthographic processing such that the information extracted from identical previews facilitated the processing of the target word, whereas extracting information from previews that do not share orthographic information with the target resulted in processing costs. The results show that the significant processing costs for Unrelated previews were observable as early as first fixation duration, and persisted into later processing measures.

### Parafoveal ortho-morphological processing

The reported results also replicate the findings from various experimental paradigms where transposing the letters of a Semitic root morpheme resulted in significant disruption to target word processing (e.g., [33,39–41,68,103]). Indeed, both TL preview conditions, despite preserving all root (and word) letter identities, yielded significantly inflated fixation durations relative to the Identity previews.

Importantly, the results suggested that the disruption to target word processing was greater for TL previews that created a new real roots. The TL New Root previews yielded significantly longer fixation durations relative to TL Pseudo Root previews. Furthermore, and whereas the processing costs associated with the TL Pseudo Root condition did not differ significantly from those of the Unrelated preview condition, the costs associated with the TL New Root preview significantly exceeded the costs of the Unrelated preview condition. The costs associated with both TL preview conditions appeared as early as first fixation duration and persisted into later processing measures. The same applies to the significant costs obtained from the TL New Root condition compared to the TL Pseudo Root condition.

These results lend strong support to the idea that lexical organization in Semitic languages is root- and not orthography-based, with root-sharing words clustering close together in the lexicon (e.g., [15,24,25,28,29,39]). Thus, the activation of the new root, created by transposing the letters of the original root in the target, led to a costly competition that inflated processing time on the target word and delayed its identification. Additionally, and given that in both TL conditions the preview strings shared all letter identities with the target, the results reported here further support the idea that the degree to which readers benefit from orthographic similarity is contingent upon the morphological characteristics of the language being read (see e.g., [15,29]), and the degree to which these properties of the linguistic environment permit flexibility, or demand rigidity, in letter position coding.

### Parafoveal morphological processing

The reported results also replicated the preview benefit obtained from previews that preserve the root morpheme information (e.g., [28,65]). The results also replicate findings from investigations using single word tasks (e.g., [2,24,25,27,43]) where primes that shared the root of the target word resulted in processing benefit for the target word relative to other conditions where the same number of letters was shared between the target word and the prime. In the current experiment root-preserving previews yielded comparable fixation durations to Identity previews, and a significant processing facilitation compared to Unrelated previews. These results thus further support the suggestion that lexical organization in Semitic languages is root-based. Arabic readers do indeed extract root morpheme information parafoveally, and this early access to the root information facilitates the identification of the up-coming target word. The results show that processing benefits from the Root preview condition relative to Unrelated previews appeared as early as first fixation duration and persisted into later processing measures.

By contrast, Pattern-preserving previews did not yield processing facilitation. Rather, these previews yielded significant processing cost relative to the Identity preview condition, and a comparable cost to processing as that observed for the Unrelated previews. In fact, additional contrasts revealed that Root previews resulted in significant processing facilitation relative to Pattern previews. In selecting the target words and previews, we included both nominal and verbal patterns given that both were found to yield processing benefit as primes in single word tasks (e.g., [1,2,36,43,44]). Although somewhat surprising, these results are broadly in line with the literature. It will be recalled that the benefits from pattern morpheme primes in Arabic were of more precarious nature, and were strongly affected by other variables such as stimulus onset asynchrony (SOA), as well as root productivity.

The greater benefit obtained from Root relative to Pattern previews can perhaps be explained by one (or a combination) of these two mechanisms. The first mechanism can be summarized as follows: Any small benefit obtained from pattern-preserving parafoveal preview may be obliterated given the sizable disruption to processing that results from embedding a completely new root in that preview [70]. The second possible mechanism was put forward by Boudelaa and Marslen-Wilson [44] (see also [24]). In this account, the way that root and pattern information is approached or relied upon for word identification by Arabic readers is essentially different given the ubiquitous presence of consonantal roots in Arabic print, as compared to the absence of vowel representations which make up a significant part of Arabic word patterns. Clearly, further investigation into the role pattern morphemes play in word identification is necessary in order to clarify the extent to which readers make use of them, and, importantly, to clearly delineate the time course of these processes. The ultimate goal is to use such findings to inform a more comprehensive theory of morphological processing in Semitic languages.

### Parafoveal semantic processing

The results reported expanded our knowledge of parafoveal processing of Arabic and suggests that Arabic readers extract semantic information from the parafovea. Synonym previews yielded comparable fixation durations to Identity previews, and a significant processing facilitation compared to Unrelated previews. As far as we are aware, this is the first time semantic preview benefit is reported in reading Arabic. This benefit appeared as early as first fixation duration and persisted into later processing measures (similar pattern of results in English reported by Schotter [80]). These results also complement findings from previous research that documented that Arabic readers show semantic priming benefit (e.g. [46,47]). We will forward two accounts that potentially accommodate these results.

Starting with a parafoveal integration account (see, e.g., [83]), it is possible that these results point at Arabic readers accessing the semantic representation of the preview, and the integration between the semantically-identical preview and target facilitated the identification of this target once it was fixated. This facilitation from the Synonym previews is not likely to be attributable to the shared orthographic representation with the target (the number of shared letters), or the shared pattern morpheme letters with the target word either. This is because the Synonym previews do not share more letters with the target relative to other preview conditions (see Table 1), and also, as the results show, sharing pattern letters with the target did not result in any preview benefit. Similarly, given that the Synonym previews did not share root information with the target, root-based facilitation of target word identification can be ruled out. It is also worth noting that the obtained benefit from the synonymous previews was not facilitated by sentential contextual constraints, that is, predictability, given that the target word and its previews were all unpredictable from previous sentence context, as we established during the sentences’ norming.

A second plausible account is potential faciliatory effect of contextual fit of the Synonym preview, that is, its plausibility (we wish to thank an anonymous reviewer for their helpful comments and guidance with regards to this issue.) Specifically, the facilitation reported was because the Synonym preview is plausible in the sentence context, regardless of its semantic closeness to the target word. Previous research showed a considerable effect of preview plausibility such that plausible previews resulted in processing benefit, regardless of their semantic links with the target (e.g., [83,84]), and independently of the orthographic relatedness between the preview and target [104], especially when the previews were not predictable from the immediate preceding context ([105] also [106] for additional empirical evidence, and [107] for a review). In essence, when the upcoming word (in the parafovea) is plausible, the information extracted from it facilitates the incremental sentence processing, regardless of the relatedness between the target and the preview, hence plausible previews generate more skipping of the target (e.g., [106]), and shorter fixation duration on the target in early processing measures when it is fixated and not skipped (e.g., [83]). As we have not controlled for target and preview plausibility, the possible effects of plausibility on the reported results were not part of our *a priori* research question. Attempting to explore, post hoc, the possible effects of plausibility, we have collected plausibility ratings from 19 additional participants, from the same population, for all the preview conditions, except the TL Pseudo Word condition. The results of this activity are summarized in S2 File. The preview conditions varied significantly in their plausibility ratings (*F*(5,1128) = 287.7, *p* < .001). Post hoc Tukey test for selected pairwise contrasts revealed that with the exception of the Synonym preview condition, all other preview conditions were rated significantly less plausible than the Identity preview (all *p*s < .001, see Table A in S2 File for descriptive statistics of the plausibility ratings). Similarly, all preview conditions, including the Synonym condition, were rated more plausible than the Unrelated condition (all *p*s < .001). As such, it is not possible to rule out that the facilitation observed for the Synonym preview condition may reflect, at least to some degree, facilitation from this preview being a plausible continuation of the sentence.

Clearly, further investigation is needed to clarify if the effects reported here are due to integration of the preview and target semantic identity, or if the high plausibility of Synonym previews was sufficient to result in the reported preview benefit effects. As a speculation, however, we will suggest that the plausibility of the preview on its own was not sufficient to generate the observed facilitation for the Synonym previews. To begin with, similar to the Identity previews, the Synonym previews were rated as significantly more plausible than all other remaining conditions (see S2 File, all *p*s < .001). Importantly, this included the Root previews. Yet, as reported above, Synonym and Root previews yielded highly comparable facilitation patterns, the discrepancy in preview plausibility ratings between these two conditions notwithstanding. Thus, a plausible speculation is that the significant preview benefit obtained from the Synonym previews perhaps reflects both benefits, combined: Integrating the preview information with the semantically-identical target, plus the facilitation afforded by this preview being a good fit with the context. Other contrasts also indicated that preview plausibility cannot solely explain the patterns of results reported. Rather, morphological parafoveal processing (e.g., preserving or violating the root information) was the main driver of the observed effects. For instance, the Root and TL New Root conditions were rated as similarly plausible (see S2 File, *p* = .55), yet, there was a sizable cost associated with the TL New Root previews, whereas the Root previews yielded the expected preview benefit. Similarly, the Pattern previews’ significantly higher plausibility rating relative to the Unrelated and the Root previews (*p*s < .001), did not translate into a preview benefit. This perhaps can be seen as further support for the integration accounts of parafoveal processing, at least as far as processing of Semitic root morphology is concerned.

Put together, the patterns of findings reported thus far suggest that in attempting to translate text into meaning, readers of Arabic prioritize these two levels of representation, root morphology and semantic/contextual information. We return to and expand upon this suggestion below.

### Theoretical implications and future directions

To begin with, our results replicate previous findings where orthographically identical previews yield processing benefit relative to orthographically unrelated previews. These findings further support the suggestion that skilled readers extract information from upcoming, parafoveal, words [48,49,51], and readers of Arabic are no exception. In addition to extracting orthographic information from the parafovea, the results indicated that they also extract root morpheme information. The importance of Semitic roots, as the base of lexical organization and their role in word identification has long been accepted and advocated in the literature (see [93] for a review). Extracting such morphological information prior to fixating the word can be seen as additional support to models of word identification in Semitic languages that postulate early and compulsory morphemic decomposition and root identification processes (e.g., [24,32,44] also [92] for further discussion). When the root information available in the parafovea was valid, processing benefit was obtained. By contrast, inaccurate (i.e., transposed) previews of root letters resulted in delaying target word identification, especially when the root letter transposition instantiated a real root—a lexical competitor.

The results reported here further challenge models of word identification that postulate flexible letter position encoding as a universal property of the cognitive system (see above). Extensive discussion of this topic and implications for modelling word identification is available elsewhere (e.g., [15,38–41]) and need not be repeated here. An issue of equal importance, however, is that current models of reading do not provide full accounts of the role of morphology in word identification as they, predominantly, focus on single-morpheme words or assume that polymorphemic words are represented as static, whole units, in the lexicon (e.g., [108,109]). Strong and consistent empirical evidence support the idea that compulsory morphological decomposition and root identification processes take place early on during word identification (see above), and this is followed by root and pattern re-combination that allows for complete word identification [92,110]. As such, models that adopt a compositional outlook (e.g., [111]) are potentially more capable of accommodating the empirical findings. In such models, mapping of patterns of orthographic features onto their corresponding phonological and semantic representations allows the activation of the compositional meaning of the word being read. This mechanism can be the core on which models that feature fully-specified morphological processing are constructed [93]. Full construction and specification of such models will need to be informed by clearer findings concerning pattern morpheme processing, as mentioned above, and a careful accommodation of the existing robust empirical findings concerning root morpheme processing.

The findings reported here indicate that in addition to benefiting from early (parafoveal) access to root morpheme information, readers of Arabic benefit from early access to semantic information. If we adopt the parafoveal integration account of these results, this deep level of linguistic processing may have been facilitated by the relative orthographic transparency of Arabic consonants (e.g., [79,80]). It is plausible that this transparency permits earlier start of semantic processing of Arabic words. This hypothesis, however, requires further investigation in carefully designed experiments. Essentially, the transparency of consonant-phoneme correspondences in Arabic is not perfect given the absence of vowel information from Arabic print—the letter ب, for instance, will always produce the /b/ sound, however, it may not be immediately obvious to the reader if the correct pronunciation of this letter in a string should be /ba/ or /bu/. In fact, lexical ambiguity is very commonplace in Arabic text such that readers as a matter of course rely on sentence context for lexical disambiguation and word identification during text reading [45,97,112]. In essence, this forces the readers of Arabic to prioritize access to morpho-semantic information in word identification, and to be guided throughout reading by sentence context (i.e., meaning comprehension) and word-context integrative processes [93,113]. It is thus unsurprising that readers of Arabic begin extracting and processing morphological and semantic information early on in the time course of translating text to meaning. Arguably, similar conclusions can be reached for previews that are plausible and fit with sentence context (at least if they do not include a violation of the Semitic root information, as the discussion above indicated). Consequently, parafoveal previews that facilitate this processing by providing early and accurate root morpheme information, highly plausible, or identical (synonymous) semantic information with the target word, result in processing benefit in the identification of this target. Future investigations will need to further explore the variables that influence semantic parafoveal processing in Semitic languages, not least of all the role of pre-target sentence context and target plausibility.

Early access to semantic information in the course of word identification, is not a novel idea. Multiple existing models of reading include semantic feedback routes to phonology and orthography (e.g., when this route provides feedback for disambiguating the pronunciations of homophonic competitors as in the Triangle Model [111,114]. In a similar manner, other models assume the presence of a semantic-to-orthography feedback route, akin to a spelling-checking mechanism [115]. The inclusion of semantic feedback allows these models to accommodate findings which suggest that accessing the semantic representation of a letter string facilitates its identification, particularly for low-frequency letter strings [116], and for morphologically complex and/or compound words [117]. However, most of the currently available models (e.g., [114]) acknowledge that the contribution of word-based semantic processing is underspecified, much less the role of context-based semantic information utilized by readers of Arabic (and other similar Semitic languages like Hebrew, see [93]). The empirical findings reported here further exemplify the need to accommodate the involvement of semantics in early word identification, and emphasize the necessity of developing comprehensive models that fully specify the roles of semantic processing at the level of single-word, and at the level of word-context semantic integration.

Finally, and with regards to eye movements control during reading, one point concerning the pattern of word skipping data that was reported here requires a brief discussion. The word skipping probabilities reported were generally in line with the average skipping rates previously reported in Arabic reading studies [93]. Hermena and Reichle [93] suggested that the informational density of Arabic words (with too many pieces of morpho-syntactic information represented in relatively short single words, see [118]; also [92]), as well as the visual complexity of the Arabic script, combined, force Arabic readers to skip fewer words (on average 8%) compared to readers of European languages (around 30%, e.g., [49,119]). With such infrequent skipping events (almost a floor effect), it is not surprising that no statistically reliable differences between the different preview conditions were found in the measure of word skipping. Thus far, word skipping rate in Arabic has reliably indexed the effect of the physical width (spatial extent) of printed words, with wider words typically skipped significantly less than narrower ones, regardless of the number of letters encompassed in that space [94,120].

A limitation of the ambitious design adopted in this study is the relatively small number of items per condition that resulted from the number of conditions we wanted to include, and the strict matching criteria adopted. Importantly, the reported results replicated stable and well-documented findings in the literature (e.g., benefits from identity and root-preserving previews). We are currently working to replicate, further clarify, and expand upon the more novel findings reported here (e.g., semantic preview benefit).

To summarize, the reported study investigated orthographic, morphological and semantic parafoveal processing in reading Arabic. The obtained results replicated the well-established findings that readers benefit from orthographically-identical previews, and that Arabic readers benefit from preserving the Semitic root of the Arabic words in the parafoveal preview. The results did not show benefit from preserving the pattern morpheme in the preview. Furthermore, the results replicated the findings that transposing the letter order of the Semitic root in the parafoveal preview results in sizable disruption to processing, especially when the letter transposition created a new real root. Thus, the reported findings further highlight how Semitic morphology, notably the consonantal root system, plays a key role in word identification, lexical organization, and modulates the degree to which the orthographic code is relied upon during early word identification processes. Finally, the results suggested that Arabic readers extract semantic information from parafoveal previews such that preview benefit was obtained from previews that were synonymous with the target word. It is likely that the high plausibility of the synonymous previous further amplified this semantic preview benefit. Further investigation into these processes is necessary. We concluded that these results can contribute towards building more comprehensive models of word identification and reading where full accounts of morphological processing and early semantic access should be further delineated and incorporated.

## Supporting information

S1 File Final reported LMM models for all contrast matrices. The final reported LMM models for the contrast matrices in Tables 3–5.
(DOCX)

S2 File Preview plausibility ratings. The procedure and outcome of obtaining plausibility ratings for previews.
(DOCX)
