# Supplementary material for: Parafoveal processing of orthographic, morphological, and semantic information during reading Arabic: A boundary paradigm investigation
Source: PLoS One. 2021 Aug 2;16(8):e0254745. doi: 10.1371/journal.pone.0254745 (PMC8328344; doi:10.1371/journal.pone.0254745)
Supplement: S1 File — The final reported LMM models for the contrast matrices in Tables 3–5. (DOCX) [file pone.0254745.s001.docx]

**Final reported LMM models for all contrast matrices**

**Table 3. Final reported LMM models for the contrast matrix of measures with Identity preview as baseline:**

Skipping: Model = glmer (dependent_variable ~ preview_condition + (1|participant) + (1|stimulus), control = glmerControl (optimizer = "bobyqa", optCtrl = list (maxfun=12000)), data = datafile, family = binomial)

First and Single Fixation Duration: Model = glmer(dependent_variable ~ preview_condition + (1|participant) + (1|stimulus), control=glmerControl(optimizer="bobyqa",optCtrl=list(maxfun=12000)), data = datafile, family= "Gamma"(link = "identity"))

Gaze Duration: Model = glmer(dependent_variable ~ preview_condition + (1 + preview_condition | participant) + (1|stimulus), control=glmerControl(optimizer="bobyqa",optCtrl=list(maxfun=12000)), data = datafile, family= "Gamma"(link = "identity"))

Total Fixation Time: Model = glmer(dependent_variable ~ preview_condition + (1|participant) + (1|stimulus), control=glmerControl(optimizer="bobyqa",optCtrl=list(maxfun=12000)), data = datafile, family= "Gamma"(link = "identity"))

Spillover: Model = glmer(dependent_variable ~ preview_condition + (1|participant) + (1|stimulus), control=glmerControl(optimizer="bobyqa",optCtrl=list(maxfun=12000)), data = datafile, family= "Gamma"(link = "identity"))

**Table 4. Final reported LMM models for the contrast matrix of measures with Unrelated preview as baseline:**

Skipping: Model = glmer (dependent_variable ~ preview_condition + (1|participant) + (1|stimulus), control = glmerControl (optimizer = "bobyqa", optCtrl = list (maxfun=12000)), data = datafile, family = binomial)

First and Single Fixation Duration: Model = glmer(dependent_variable ~ preview_condition + (1|participant) + (1|stimulus), control=glmerControl(optimizer="bobyqa",optCtrl=list(maxfun=12000)), data = datafile, family= "Gamma"(link = "identity"))

Gaze Duration: Model = glmer(dependent_variable ~ preview_condition + (1|participant) + (1|stimulus), control=glmerControl(optimizer="bobyqa",optCtrl=list(maxfun=12000)), data = datafile, family= "Gamma"(link = "identity"))

Total Fixation Time: Model = glmer(dependent_variable ~ preview_condition + (1|participant) + (1|stimulus), control=glmerControl(optimizer="bobyqa",optCtrl=list(maxfun=12000)), data = datafile, family= "Gamma"(link = "identity"))

Spillover: Model = glmer(dependent_variable ~ preview_condition + (1|participant) + (1|stimulus), control=glmerControl(optimizer="bobyqa",optCtrl=list(maxfun=12000)), data = datafile, family= "Gamma"(link = "identity"))

**Table 5. Final reported LMM models for the additional pre-specified contrasts:**

Skipping: Model = glmer (dependent_variable ~ preview_condition + (1|participant) + (1|stimulus), data = datafile, family = binomial)

First Fixation Duration: Model = Model = glmer(dependent_variable ~ preview_condition + (1 + preview_condition | participant) + (1|stimulus), control=glmerControl(optimizer="bobyqa", optCtrl=list(maxfun=12000)), data = datafile, family= "Gamma"(link = "identity"))

Single Fixation Duration: Model = Model = glmer(dependent_variable ~ preview_condition + (1| participant) + (1|stimulus), control=glmerControl(optimizer="bobyqa", optCtrl=list(maxfun=12000)), data = datafile, family= "Gamma"(link = "identity"))

Gaze Duration: Model = glmer(dependent_variable ~ preview_condition + (1|participant) + (1|stimulus), data = datafile, family= "Gamma"(link = "identity"))

Total Fixation Time: Model = glmer(dependent_variable ~ preview_condition + (1|participant) + (1|stimulus), control=glmerControl(optimizer="bobyqa",optCtrl=list(maxfun=12000)), data = datafile, family= "Gamma"(link = "identity"))

Spillover: Model = glmer(dependent_variable ~ preview_condition + (1 + preview_condition | participant) + (1|stimulus), control=glmerControl(optimizer="bobyqa",optCtrl=list(maxfun=12000)), data = datafile, family= "Gamma"(link = "identity"))
