# Supplementary material for: Parafoveal processing of orthographic, morphological, and semantic information during reading Arabic: A boundary paradigm investigation
Source: PLoS One. 2021 Aug 2;16(8):e0254745. doi: 10.1371/journal.pone.0254745 (PMC8328344; doi:10.1371/journal.pone.0254745)
Supplement: S2 File — The procedure and outcome of obtaining plausibility ratings for previews. (DOCX) [file pone.0254745.s002.docx]

**S2 Discussion.**

**Preview plausibility ratings**

Nineteen additional participants from the same population group rated the plausibility of the previews on a scale that ranged from 1 = highly implausible to 7 = highly plausible. The participants saw any of the experimental sentences only once, in one of the preview conditions. The preview condition TL Pseudo Root was not included given that the root letter transposition created a non-word. The output is below in Table S4.

**Table A. Descriptive statistics of plausibility ratings of all preview conditions (excl. TL Pseudo Root)**

|  | Preview Condition | | | | | |
| --- | --- | --- | --- | --- | --- | --- |
|  | Identity | Pattern | Root | Synonym | TL New Root | Unrelated |
| Average Plausibility Rating (SD) | 5.7  (1.1) | 4.0  (1.9) | 2.6  (1.7) | 5.4  (1.2) | 2.9  (1.8) | 1.1  (0.3) |
